# 99 Empowering Emergency Responders to Treat on Scene Alleviates Hospital Strain During Disaster Events

**DOI:** 10.1093/jbcr/irae036.098

**Published:** 2024-04-17

**Authors:** Anastasiya Ivanko, Jonathan E Schoen, Elizabeth Lacy, Carl A Flores, Randy D Kearns, Herb A Phelan, Jeffrey E Carter

**Affiliations:** University Medical Center (LSU Health), New Orleans, LA; University Medical Center New Orleans, LAPLACE, Louisiana; University Medical Center New Orleans, New Orleans, Louisiana; University of New Orleans, New Orleans, LA; Louisiana State University Health Sciences Center- New Orleans, New Orleans, LA; University Medical Center (LSU Health), New Orleans, LA; University Medical Center New Orleans, LAPLACE, Louisiana; University Medical Center New Orleans, New Orleans, Louisiana; University of New Orleans, New Orleans, LA; Louisiana State University Health Sciences Center- New Orleans, New Orleans, LA; University Medical Center (LSU Health), New Orleans, LA; University Medical Center New Orleans, LAPLACE, Louisiana; University Medical Center New Orleans, New Orleans, Louisiana; University of New Orleans, New Orleans, LA; Louisiana State University Health Sciences Center- New Orleans, New Orleans, LA; University Medical Center (LSU Health), New Orleans, LA; University Medical Center New Orleans, LAPLACE, Louisiana; University Medical Center New Orleans, New Orleans, Louisiana; University of New Orleans, New Orleans, LA; Louisiana State University Health Sciences Center- New Orleans, New Orleans, LA; University Medical Center (LSU Health), New Orleans, LA; University Medical Center New Orleans, LAPLACE, Louisiana; University Medical Center New Orleans, New Orleans, Louisiana; University of New Orleans, New Orleans, LA; Louisiana State University Health Sciences Center- New Orleans, New Orleans, LA; University Medical Center (LSU Health), New Orleans, LA; University Medical Center New Orleans, LAPLACE, Louisiana; University Medical Center New Orleans, New Orleans, Louisiana; University of New Orleans, New Orleans, LA; Louisiana State University Health Sciences Center- New Orleans, New Orleans, LA; University Medical Center (LSU Health), New Orleans, LA; University Medical Center New Orleans, LAPLACE, Louisiana; University Medical Center New Orleans, New Orleans, Louisiana; University of New Orleans, New Orleans, LA; Louisiana State University Health Sciences Center- New Orleans, New Orleans, LA

## Abstract

**Introduction:**

Disaster events can cause prolonged losses of electricity, leaving individuals to utilize alternative sources of energy such as fossil fuel generators, which in turn can lead to burn injuries and/or carbon monoxide (CO) exposure. The purpose of this study was to examine the impact of generator-related burn injury and CO poisoning (COP) following a region-wide disaster, and the effects of empowering EMS providers to treat many of these injuries on-scene.

**Methods:**

Utilizing data gathered from Emergency Medical Services (EMS) provider agencies, we constructed three cohorts related to Hurricane Ida’s landfall: PRE-IDA (07/08/21 - 08/25/21), MID-IDA (08/26/21 – 09/08/21) and POST-IDA (09/09/21 – 10/31/21). We analyzed EMS calls, dispatches, and transports for primary complaints indicating COP or burn injury during these time periods. Statewide emergency response network representatives were contacted regarding and pre-hospital care and coordination during disaster responses.

**Results:**

PRE-IDA EMS averaged 15.4 calls per day indicating COP or burn injury. 1.4 (9%) of these calls were due to burn injury (61% of which were transported to nearest ED), while 13 (91%) were due to suspicion of COP (98% of which were transported to nearest ED). The mean turnaround time from dispatch to EMS being back in service was 63 (±36) minutes. MID-IDA EMS averaged 20.9 calls per day. 3.3 (16%) of these calls per day were due to burn injury, and 16.7 (84%) of these calls per day were due to COP. Of the MID-IDA calls, 73% of burn patients and 78% of COP patients were transported to the nearest ED. The mean turnaround time from dispatch to EMS being back in service was 64 (±31) minutes during MID-IDA. During POST-IDA, the mean calls per day to EMS decreased to 10.6 (16% of which were burn injury, 84% of which were suspected COP). Of the POST-IDA dispatches, 61% of burn patients and 97% of COP patients were transported to the nearest area hospital. The mean turnaround time from dispatch to EMS being back in service was 69 (± 49) minutes. Lastly, there were no fatalities from COP in patients transported to the burn center and only one patient required hyperbaric therapy for isolated COP.

**Conclusions:**

At the peak, EMS had 90 calls each day per parish for COP requiring implementation of disaster plan. Our data shows an increase in CO related EMS dispatches during MID-IDA with fewer patients transported to regional area hospitals, but overall stable transport times from household to area hospitals amongst the three time periods. This study demonstrates the collaboration of a verified burn center and the EMS community when responding a to natural disaster.

**Applicability of Research to Practice:**

This study demonstrates that empowering EMS to treat smaller burn injuries, and clinically mild CO exposures, on scene during a disaster event decreases strain on area hospitals’.

